# Sharing More than Friendship — Nasal Colonization with Coagulase-Positive Staphylococci (CPS) and Co-Habitation Aspects of Dogs and Their Owners

**DOI:** 10.1371/journal.pone.0035197

**Published:** 2012-04-18

**Authors:** Birgit Walther, Julia Hermes, Christiane Cuny, Lothar H. Wieler, Szilvia Vincze, Yassmin Abou Elnaga, Ivonne Stamm, Peter A. Kopp, Barbara Kohn, Wolfgang Witte, Andreas Jansen, Franz J. Conraths, Torsten Semmler, Tim Eckmanns, Antina Lübke-Becker

**Affiliations:** 1 Institute of Microbiology and Epizootics, Veterinary Faculty, Freie Universität Berlin, Germany; 2 Department for Infectious Disease Epidemiology, Robert Koch Institute, Berlin, Germany; 3 National Reference Centre for Staphylococci, Robert Koch Institute, Wernigerode Branch, Germany; 4 Vet Med Labor GmbH, Division of IDEXX Laboratories, Ludwigsburg, Germany; 5 Clinic of Small Animals, Veterinary Faculty, Freie Universität Berlin, Berlin, Germany; 6 Institute of Epidemiology, Friedrich-Loeffler-Institut, Federal Research Institute for Animal Health, Wusterhausen, Germany; The University of Hong Kong, Hong Kong

## Abstract

**Background:**

Since the relationship between dogs and their owners has changed, and dogs moved from being working dogs to family members in post-industrial countries, we hypothesized that zoonotic transmission of opportunistic pathogens like coagulase positive staphylococci (CPS) is likely between dogs and their owners.

**Methodology/Principal Findings:**

CPS- nasal carriage, different aspects of human-to-dog relationship as well as potential interspecies transmission risk factors were investigated by offering nasal swabs and a questionnaire to dog owners (108) and their dogs (108) at a dog show in 2009. *S. aureus* was found in swabs of 20 (18.5%) humans and two dogs (1.8%), and *spa* types which correspond to well known human *S. aureus* lineages dominated (e.g. CC45, CC30 and CC22). Multilocus sequence typing (MLST) of the two canine strains revealed ST72 and ST2065 (single locus variant of ST34). Fifteen dogs (13.9%) and six owners (5.6%) harboured *S. pseudintermedius*, including one *mec*A-positive human isolate (MRSP). Pulsed field gel electrophoresis (PFGE) revealed that one dog/owner pair harboured indistinguishable *S. pseudintermedius-* isolates of ST33. Ten (48%) of the 21 *S. pseudintermedius*-isolates showed resistance towards more than one antimicrobial class. 88.9% of the dog owners reported to allow at least one dog into the house, 68.5% allow the dog(s) to rest on the sofa, 39.8% allow their dogs to come onto the bed, 93.5% let them lick their hands and 52.8% let them lick their face. Bivariate analysis of putative risk factors revealed that dog owners who keep more than two dogs have a significantly higher chance of being colonized with *S. pseudintermedius* than those who keep 1–2 dogs (p<0.05).

**Conclusions/Recommendations:**

In conclusion, CPS transmission between dog owners and their dogs is possible. Further investigation regarding interspecies transmission and the diverse adaptive pathways influencing the epidemiology of CPS (including MRSA and MRSP) in different hosts is needed.

## Introduction

In post-industrial countries, the socio-economic relationship between many dogs and their owners has changed dramatically during the last decades [1]. Instead of having working dogs (watch dogs, sheepdogs etc.) living in stables or kennels, people keep pets who live in the household almost as family members. In particular people who feel disconnected from society tend to substitute social contacts by pets, including supportive anthropomorphic traits (“humanisation" of non-human beings) [2], [3]. As a result of this development, transmission of microorganisms between humans and their dogs may increase [4]. Especially microorganisms that reside on the skin or mucosal surfaces of dogs might easily be transmitted to pet owners either by direct contact or by sharing the same environment in the household, e.g. sofas, beds and bath tubs.

Commensals usually consist of a wide range of different bacterial species, including coagulase-positive staphylococci (CPS) like *S. aureus* and *S. pseudintermedius*. While *S. aureus* is a common colonizer in humans and widely distributed among mammalian hosts in general, *S. pseudintermedius* seems to be predominantly associated with small animals (i.e. dogs, cats) and was found only occasionally in human specimens in the past [5], [6], [7], [8].

Opportunistic pathogens like CPS seem to be of special interest, since reports about the general transferability of these bacteria between pets and humans have increased during recent years, including methicillin resistant variants of *S. aureus* (MRSA) and S. *pseudintermedius* (MRSP) [9], [10], [11], [12], [13], [14].

MRSA have evolved to one of the major nosocomial pathogens in healthcare systems, causing an increased mortality risk for patients and enormous costs for health insurance companies worldwide [15]. In the field of veterinary medicine, nosocomial outbreaks caused by MRSA have recently gained attention, e.g. in equine and small animal clinics [16], [17]. Nasally colonized veterinary personnel seem to have an impact on rising infection rates in animal patients, just like health care workers in hospitals [14].

As far as CPS are concerned, *S. pseudintermedius* seems to be more common among dogs and cats than *S. aureus* [10]. Moreover, certain strains of *S. pseudintermedius* have become a major threat in veterinary medicine due to their frequent methicillin- and multidrug resistant appearance [18], [19].

The first serious human infections with *S. pseudintermedius* (including MRSP) have recently been reported [20], [21]. Owners of dogs suffering from dermatitis seem to be frequently colonized with *S. pseudintermedius* strains (including MRSP) [13], [22], [23].

In general, data about the colonization of dogs and their owners with CPS in the community are scarce. Hanselman *et al.* (2009) reported that *S. aureus*, *S. pseudintermedius* and *S. schleiferi* ssp. *coagulans* (including methicillin-resistant variants) were harboured by dogs living in households. In addition, concurrent human and animal colonization by indistinguishable *S. aureus* and *S. pseudintermedius* strains have been observed [10]. Against this backdrop, the question has arisen how changes in the human-to-dog relationship influence inter-species CPS transmission.

Therefore, the aim of this study was to assess the nasal colonisation of dogs and their owners with CPS and to gain insights into potential risk factors for CPS transmission among them.

## Materials and Methods

### Questionnaires and ethical considerations

The study procedure and protocols were based on informed consent of the participants.

The sampling procedures in dogs had been approved by the Ethical Committee of the veterinary department of the Free University of Berlin (date: 03.03.2009).

Sampling of dog owners was approved by the Ethical Committee of the Charité, Campus Virchow-Clinic (date: 10.10.2008, Berlin).

After the dog owners had been informed about the study, they were given a questionnaire to collect demographic data on owners (e.g. gender, age and profession), number and keeping conditions of the dogs and information on the quality of contacts they shared (e.g. frequency and type of dog handling, including physical contact). Questionnaires were completed by the owners just before or after the nasal swabs were taken.

### Sampling procedure and bacterial growth

Nasal swabs (MASTASWAB™; MAST Diagnostica GmbH, Rheinfeld Germany), were simultaneously collected from voluntarily participating pet owners (by physicians) and dogs (by veterinarians) as a convenience sample on a single day at a dog show event in Berlin in March 2009.

Until processing, all nasal samples were stored for approximately 36 h at 4°C. Swabs were plated directly on columbia agar containing 5% sheep blood and BBL™ CHROMagar™ MRSA II (Becton Dickinson GmbH, Heidelberg, Germany). All incubated plates were investigated twice, first after 18 h and a second time after 36 h of incubation at 37°C. CPS suspected colonies were picked (at least two per plate) and subcultured.

### Identification and typing of isolates

Phenotypic characterisation of CPS was performed as previously reported [8], [24]. Briefly, staphylococcal isolates were identified as CPS based on colony morphology, a positive catalase reaction, gram stain appearance and a positive tube coagulase reaction. All CPS were further screened for *mec*A by PCR and *nuc* for species verification in case of *S. aureus* [25]. Species verification of *S. pseudintermedius* was performed by the MboI restriction analysis method of *pta* as described before [26].

Automated antimicrobial susceptibility testing of *S. pseudintermedius* was performed by Vet Med Labor GmbH (Ludwigsburg, Germany) using the bioMerieux VITEK®2 system according to the manufacturer's instructions including penicillin, ampicillin-sulbactam, oxacillin, gentamicin, kanamycin, enrofloxacin, marbofloxacin, erythromycin, clindamycin, tetracycline, nitrofurantoin, chloramphenicol and trimethoprim- sulfamethoxazole, following the CLSI guidelines.

All *S. aureus* strains underwent *spa*-typing and PCR detection of Panton-Valentine leukocidin (*luk*S-PV, *luk*F-PV) as previously reported [27], [28]. MLST was performed on canine *S. aureus* strains as described [29]. Possible associations between *spa* types and corresponding clonal complexes (CC) were determined according to the Ridom (http://spaserver.ridom.de/) and the MLST database (www.mlst.net).

MLST analysis on *S. pseudintermedius* was performed according to a scheme published by Bannoehr *et al.* (2007) including *pta*, *cpn*60, *tuf*, 16S rRNA and *agr*D [30]. Allele numbers and new STs were assigned according to the curator of the MLST database V. Perreten (vincent.perreten@vbi.unibe.ch).

PFGE analysis on *S. pseudintermedius* was carried out using endonuclease SmaI according to Ruscher *et al.* 2010 [19]. PCR-based detection of the bicomponent leukotoxin Luk-I (*luk*S and *luk*F) was carried out as described previously [31]. The presence of the exfoliative toxin ExpA (formerly named EXI) was determined by PCR with the primers *exp*A-F GCGCGTCCTTCTGATCCAGAACT and *exp*A-R AACGTCCCCCTTTACCTACGTGAAT (according to AB489850.1, [32] at an annealing temperature of 58°C (25 cycles) including strain IMT21652 (JN604832) as a positive control. Detection of the gene encoding ExpB was performed as described previously [33].

### Statistical analysis

Statistical analysis was conducted using STATA, version 11 (StataCorp). For bivariate analysis of risk factors for human CPS colonization, Odds Ratios (OR) were calculated and potential associations checked for statistical significance using the 2-tailed Fisher exact test. A value of p<0.05 was considered significant. Results were adjusted for age and sex.

## Results

### Description of study population

In total, 108 dog/owner pairs participated in this study. All owners completed the questionnaire and permitted nasal screening, resulting in 108 swabs of human and 108 of canine origin. Blank information with regard to individual questions (unanswered questions) was marked and excluded from statistical analysis of risk factors.

The mean age of the study participants was 48.5 years (range: 23–87 years, median 49 years), 75% were female and all but one were German residents. Analysing the zip codes (first three digits) of the places where the dog owners lived revealed a wide geographic distribution over Germany (data not shown). 50.9% of the participants declared to own only one dog, while 47.2% of them possessed two or more dogs (median: one dog, mean 2.2 dogs, range 1–11 dogs). Nine of the dog owners worked in a healthcare associated area (hospital, nursing home, medical practise) and three were veterinarians. On the day of sampling, only one dog that accompanied the owner was included in the study.

### Behavioural aspects of human-pet cohabitation

The majority of the study subjects (96/108; 88.9%) reported to share their habitation with one or more dogs, while ten (9.3%) kept the dogs in a kennel or garden. Seventy-four dog owners (68.5%) allowed their dogs to rest on the sofa, 43 (39.8%) permitted them to come onto their beds, 101 (93.5%) let their dogs lick their hands, 57 (52.8%) allowed them to lick their face, and 35 (32.4%) reported washing their dogs in their own bath tubs (Table 1).

**Table 1:** Descriptive characteristics of dog owners.

	Answers					
	positive		negative		no data	
Dog owner characteristics (n = 108)	No.	%	No.	%	No.	%
**Sex**					0	
Female	81	75.0				
Male	27	25.0				
**Keeps dog/dogs in the house**	96	88.9	10	9.3	2	1.9
**Dog breeding**	22	20.4	80	74.1	6	5.6
**Number of dogs in household**					2	1.9
one dog	55	50.9				
two dogs	25	23.1				
three or more dogs (range: 3 to 11)	26	24.1				
**Allows to lick the hands (total)**	101	93.5	5	4.6	2	1.9
**Allows to lick the face (total)**	57	52.8	37	34.3	14	13.0
**Washes dog in bath tub (total)**	35	32.4	53	49.1	20	18.5
**Allows dog to rest on sofa (total)**	74	68.5	28	25.9	6	5.6
**Allows dog to rest on bed (total)**	43	39.8	53	49.1	12	11.1

### *S. aureus* nasal carriage in healthy dogs and their owners and strain characterisation

Nasal carriage of *S. aureus* was found in 20 (18.5%) dog owners and two dogs (1.8%). With regard to *S. aureus-*positive individuals, no simultaneous colonization of dog and dog owner was observed in this study. One of the three veterinarians and one of the nine participants with a known healthcare association were found to be *S. aureus* carriers. None of the *S. aureus*-isolates harboured the genes encoding for PVL or methicillin resistance.

*Spa-*typing of all 22 *S. aureus* isolates showed a broad heterogeneity and wide distribution, including two new *spa* variants. According to the *spa* database, the twenty *S. aureus* isolates of human origin showed the following results: Four strains were assigned to *spa* types which presumptively correspond to CC45, three strains to CC59 and three others to CC7. Two strains may correspond to CC30 and two more to CC22. Another six *spa* types t346, t495, t156, t1294, t8641 (new) and t8640 (new) remained unassigned (Table 2).

**Table 2:** Molecular characteristics of *S. aureus* from the nasal cavity of dog owners and dogs.

Strain ID^1^	Pair ID^2^	Host	*mec*A	*spa* 3	MLST/CC^4^
IMT18913	2	human	neg.	t216	CC59*
IMT18916	10	human	neg.	t005	CC22*
IMT18787	11	human	neg.	t005	CC22*
IMT18788	12	human	neg.	t346	n.p.
IMT18790	16	human	neg.	t495	n.p..
RKI-09-01583	19	human	neg.	t156	n.p.
IMT18876	29	human	neg.	t350	CC45*
IMT18917	30	human	neg.	t015	CC45*
IMT18795	47	human	neg.	t289	CC7*
IMT18806	48	human	neg.	t289	CC7*
IMT18805	50	human	neg.	*t8641*	n.p.
IMT18808	52	human	neg.	t091	CC7*
IMT18809	58	**dog**	neg.	t153	**ST2065**
RKI-09-01595	62	human	neg.	t015	CC45*
IMT18826	66	human	neg.	t216	CC59*
IMT18829	88	human	neg.	t1294	n.p.
IMT18832	89	human	neg.	t275	CC30*
IMT18874	102	human	neg.	*t8640*	n.p.
IMT18887	104	human	neg.	t5201	CC59*
IMT18888	109	**dog**	neg.	t5199	**ST72**
IMT18872	110	human	neg.	t5200	CC30*
IMT18873	111	human	neg.	t015	CC45*

1 Strain collection number: IMT: Institute of Microbiology and Epizootics, RKI: Robert Koch-Institute (Wernigerode branch).

2 Individual number for each of the participating dog/dog owner pairs.

3 *italic:* new *spa* types.

4 ST determined according to MLST result (bold),

* predicted CC according to (www.ridom.de), n.p.: not predictable.

MLST-analysis of the two canine *S. aureus*-isolates revealed that one belonged to the genetic lineage ST72 (IMT18888), and the other to ST2065 (IMT18809), a single locus variant of ST34 (Table 2: bold letters).

### S. *pseudintermedius* nasal carriage in healthy dogs and their owners and strain characterisation

Six owners (5.6%) and fifteen dogs (13.9%) were carriers of *S. pseudintermedius*. In addition, one of the six human isolates (IMT18885) proved to be *mec*A-positive (MRSP). None of them were veterinarians or healthcare workers.

**Figure 1:**
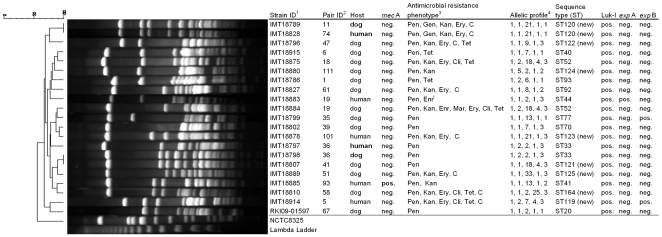
Comparative PFGE analysis and strain characteristics of *S. pseudintermedius* from human and canine origin. Dendrogram (percent similarity) showing DNA restriction pattern after digestion with *Sma*I for all 21 MSSP and MRSP isolates. PFGE analysis by use of bionumerics® (unweighted-pair group method using average linkages), dice coefficient, 1.2% tolerance and 0.5% optimization. Pair 36 comprised the isolates IMT18797 (canine) and IMT18798 (human), which show indistinguishable PFGE pattern. The canine isolate IMT18789 and IMT18828 (human) share an indistinguishable pulsotype as well, although they did not compose a dog/dog owner pair. ^1^Strain collection number: IMT: Institute of Microbiology and Epizootics. RKI: Robert Koch-Institute (Wernigerode branch). ^2^Individual number for each of the participating dog/dog owner pairs. ^3^Determined by use of VITEK® 2 (bioMerieux) according to the manufacturers instructions. ^4^Allelic profile of the genes *pta*, *cpn*60, *tuf*, 16S rRNA and *agr*D. Abbreviations: PEN, Penicillin G; GEN, Gentamicin; KAN, Kanamycin; ENR, Enrofloxacin; MAR, Marbofloxacin; ERY, Erythromycin; CLI, Clindamycin; TET, Tetracycline; CHL, Chloramphenicol; i: susceptibility testing result: intermediate. Luk-I: Leukotoxin I (l*uk*S and *luk*F), *exp*A: Exfoliative toxin A (primer: this study), *exp*B: Exfoliative toxin B.

MLST analysis of all 21 *S. pseudintermedius* isolates of human and canine origin revealed a heterogenic distribution of already known and 9 new sequence types (Fig. 1).

Concurrent owner and dog *S. pseudintermedius*-colonization was demonstrated for the isolates IMT18797 (human) and IMT18798 (canine) by PFGE pattern analysis, revealing one pulsotype for both isolates, which belongs to ST33 (Fig. 1, pair 36**).** Two further *S. pseudintermedius*-isolates had indistinguishable PFGE patterns but were obtained from unrelated canine and human individuals of pair 11 and 74, respectively (Fig. 1; IMT18789 and IMT18828).

All 21 *S. pseudintermedius* isolates were positive for LukI, one yielded a positive signal for ExpA and two for ExpB (Fig. 1).

Each of the 21 *S. pseudintermedius* isolates showed at least penicillin resistance (100%), while 12 (57%) were resistant towards kanamycin, 10 (48%) to erythromycin, 8 (38%) to chloramphenicol, 7 (33%) to tetracycline, 4 (19%) to clindamycin, 2 (9%) to gentamicin, one (4%) to enrofloxacin and one (4%) to marbofloxacin. The isolates were susceptible to the remaining antimicrobials (Fig. 1).

### *S. schleiferii* ssp. *coagulans* nasal carriage in healthy dogs and their owners

A single strain of *S. schleiferii* ssp. coagulans (IMT18886) was isolated from the nostrils of one dog. This isolate was not further analysed.

### Statistical analysis of potential risk factors associated with *S. pseudintermedius* carriage in humans

Dog owners who kept more than two dogs had a significantly higher chance of being colonized with *S. pseudintermedius* than those who kept 1–2 dogs (p<0.05). No other suspected risk factor proved statistically significant, but elevated chances for *S. pseudintermedius* colonization were observed for dog breeders (OR = 3.9), for owners who allowed dogs to lick their faces (OR = 3.5), to rest on the sofa (OR = 2), or to sleep in their bed (OR = 1.25). A summary of the risk factor analysis is provided in Table 3. The result remained unchanged when adjusted for age and sex.

**Table 3:** Risk factors for *S. pseudintermedius* colonization among dog owners sampled during a dog show event in Berlin in 2009 (n = 108) (bivariate analysis).

Exposure	Valid response		Nasal colonisation state of dog owners			Bivariate analysis (2-sided Fisher's exact test)	
			*S. pseudintermedius*	*S. pseudintermedius*			
			positive	negative			
	no (%)		no (% of valid responses)	no (% of valid responses)	OR	p-value	95% CI
Sex	108 (100%)	f	4 (66.6%)	77 (75.5%)			
		m	2 (33.3%)	25 (24.5%)	1.54	0.6	0.1–11.3
Keeps dog/s in the house	106 (98.1%)		6 (100%)	90 (90.0%)	-*	1	0.2 -
Breeds dogs	102 (94.4%)		2 (50.0%)	20 (20.4%)	3.9	0.2	0.3–55.9
Keeps >2 dogs	106 (98.1%)		5 (100%)	21 (20.8%)	-*	0.0006	4.7 -
Dog/s allowed to lick the face	94 (87.0%)		5 (83.3%)	52 (59.1%)	3.5	0.4	0.4–168.2
Dog/s allowed to lick hands	106 (98.1%)		6 (100%)	95 (95.0%)	-*	1	0.07 -
Dog/s in bath tub	88 (81.5%)		1 (20.0%)	34 (41.0%)	0.4	0.6	0.0–3.9
Dog/s allowed on sofa	102 (94.4%)		5 (83.3%)	69 (71.9%)	2	1	0.2–95.9
Dog/s allowed in bed	96 (88.9%)		3 (50.0%)	40 (44.4%)	1.25	1	0.2–9.8

Abbreviations:

f, female; m, male;

* not calculated.

OR: Odds ratio.

CI: Confidence interval.

## Discussion

This study aimed at elucidating aspects of the relationship and behavioural patterns between dog owners and their pets with regard to nasal colonisation with CPS. In addition, comparative analysis of CPS-carriage among dogs and humans allowed insights into the proportions of CPS carriage for each group, with a special focus on CPS as zoonotic pathogens.

Substantial proportions of the participating dog owners not only shared their house (88.9%) with their pets, but also their sofa (68.5%) or their beds (39.8%). Many dogs had obviously gained a status nearly equal to that of a (human) family member, i.e. the animals were given privileges formerly exclusively meant for humans, which are behavioural aspects typical for anthropomorphism.

Zoonotic transmission of CPS has been described [11], [13], [34] and it appears likely that the intensive daily contact between dogs and their owners may increase the likelihood of getting colonized by interspecies-transmission of CPS for both sides.

It is noteworthy that individual refusals to answer certain questions reached up to 11%, which may have biased the study results (Tables 1 and 3, columns “no data" and “valid response"). Furthermore, almost half of the participating dog owners (47.2%) stated that they keep more than just the one dog that was available for sampling on the day of sampling. While the CPS-colonisation status of the other dog(s) remains unknown, it can be assumed that all dogs of one owner are likely to live in similar co-habitation conditions.

Longitudinal studies regarding *S. aureus* nasal carriage in humans report approximately 20% (range 12–30%) of individuals who are persistently colonized and 50% (range 16–69%) non-carriers depending on the study protocols and methods (e.g. use of enrichment broth, kind of study population) [35]. A recent study demonstrated 19.1% nasal colonisation in 278 healthy humans in Spain [36]. Eventually, the observed 18.5% *S. aureus*-colonization in the human participants of our study is marginally lower but in overall agreement with formerly published results and probably reflecting the abandonment of enrichment broth.

At present, data on the colonization of healthy dogs with *S. aureus* gained in a community environment is very limited. In this study, 1.9% nasal *S. aureus*- carriage was found among those dogs. When studies on *S. aureus* nasal carriage in dogs are compared, care should be taken with regard to individual study backgrounds, as the kind and selection of study participants, the use of enrichment broth, sampling in a clinical or in a non-clinical environment must be expected to influence the study results. However, Rubin *et al.* found 13 of 167 (7.8%) nasal *S. aureus*- colonization among clinically healthy dogs presented for vaccination in a veterinary college [37], while Fazakerley *et al.* reported 2.3% (1 of 43) nasal colonization in healthy dogs [38]. In addition, we reported about *S. aureus-* (nasal) carriage of 5.7% in dogs on admission to an animal hospital [14].

S*pa*-typing of all human and canine *S. aureus* revealed a high diversity of *spa* types as previously observed for community associated *S. aureus* [36]. The majority of *spa* types of human isolates (16/20) seemed to be associated with well known human lineages (e.g. CC45, CC30, CC22) and the remaining six had not been described in any context to animal associated lineages before. Therefore, a risk factor analysis for *S. aureus-*carriage in humans could not be conducted.

Interestingly, one of the two canine MSSA belonged to ST72, a genetic background also displayed by the USA700 type strain (CA-MRSA) and further MSSA and MRSA of human origin (http://saureus.mlst.net). The second canine strain is a single nucleotide variant of ST34, an ST that had also previously been associated with MRSA of human origin [39]. In conclusion, both canine *S. aureus* strains isolated in this study may have once originated from a human host. These strains could belong to extended host spectrum genotypes (EHSGs), which were previously reported for MRSA strains of equine origin [40]. Consequently, dogs can be colonized with EHSG *S. aureus* strains (including MRSA) and represent a potential source of (re-) infection for humans [11].

Surprisingly, 5.5% of dog owners were found to be positive for S. *pseudintermedius* nasal carriage, including MRSP in one case. A recent study by Paul and co-workers found a nasal colonisation in 3.9% of 128 veterinarians attending a conference for animal dermatologists [41]. All strains were proven to be MRSP and their nasal carriage persisted for at least one month. While human infections with *S. pseudintermedius* have occasionally been reported in the past, an increased awareness of these CPS in human medicine ensued very recently because of the multi-drug resistant phenotype which is frequently associated with MRSP [19], [41], [42]. We found one dog/owner pair contemporarily colonized with one particular *S. pseudintermedius* strain, as has previously been reported for MRSP in clinical cases [13], [43]. Moreover, another person and a dog who had no relationship according to their pair ID's were colonized with strains displaying an indistinguishable PFGE-pattern, which emphasizes the transferability of *S. pseudintermedius*.

While *S. pseudintermedius* is considered as a common colonizer in the first line [7], the antibiotic resistance phenotypes demonstrated that ten of the 21 isolates showed resistance towards four or more antimicrobials. Among these isolates was strain IMT18884, which showed resistance towards 7 agents (Fig. 1).

It is striking that all six dog owners who carried *S. pseudintermedius* kept their dogs in the house, and had three or more dogs (one person did not answer the respective question). Statistical analysis supports the result by revealing that keeping two or more dogs is significantly associated with *S. pseudintermedius* colonization in humans (p = 0,0006). Other suspected risk factors were not statistically significantly associated with *S. pseudintermedius* carriage in our analysis. Interpretation of statistical results needs to take the small number of cases into account.

As a matter of fact, more dogs will result in more saliva as well as more dog scurf and hair harbouring commensals (including CPS) in the household environment shared by both, dogs and owners. In addition, domestic contamination due to *S. pseudintermedius* has been reported for households of MRSP-infected pets before, and it was assumed that dust particles may play a role in this finding [34].

Furthermore, it was thought that certain MRSP lineages (e.g. ST71) may have a greater ability to adapt to the human host [41] than MSSP. However, it is important to note that the MRSP and MSSP strains from human origin detected in this study comprised six different STs (Fig. 1). Regarding the exfoliative toxins in MSSP reported on here, one human isolate yielded a positive PCR result for ExpA, while two canine strains were found positive for ExpB, indicating that these virulence factors may be more frequently associated with cases of superficial dermatitis in dogs [32], [33].

The single MRSP (IMT18885) isolated during this study was of human origin, showed an ST41 background and was susceptible to most of the tested antimicrobials. In addition, IMT18885 had an oxacillin MIC of 2 mg/l and expressed a comparatively low MIC (4/2 mg/l) towards ampicillin-sulbactam (Fig. 1). Interestingly, this ST was originally described for a canine isolate (*mec*A negative) in the USA [30]. It seems likely that acquisition events of *mec*A harbouring staphylococcal chromosomal cassettes will occur in accessible strains over time.

In conclusion, exposure of humans to pet dogs seems to be associated with the possibility of getting colonized by *S. pseudintermedius*, regardless whether these bacteria are methicillin-resistant or not. Methicillin-resistant CPS attract more scientific interest because of the clinical relevance of MRS, but according to our findings the inter-species transferability does not seem to be necessarily linked with methicillin-resistance at all. However, it remains unknown whether *S. pseudintermedius* colonization in humans is transient or permanent. Further investigations regarding inter-species transmission and the diverse adaptive pathways influencing the epidemiology of CPS (including MRSA and MRSP) in the community are needed. Especially the close relationship between pets and their owners underline the importance of interdisciplinary research including human and veterinary microbiologists as well as epidemiologists as demonstrated by our study results.

## References

[1] Franklin A (1999). Animals and modern cultures: a sociology of human-animal relations in modernity.

[2] Blouin DD (2008). All in the Family? Understanding the Meaning of Dogs and Cats in the Lives of American Pet Owners [dissertation].

[3] Epley N, Waytz A, Akalis S, Cacioppo JT (2008). When we need a human: motivational determinants of anthropomorphism.. Social Cognition.

[4] Wieler LH, Ewers C, Guenther S, Walther B, Lübke-Becker A (2011). Methicillin-resistant staphylococci (MRS) and extended-spectrum beta-lactamases (ESBL)-producing Enterobacteriaceae in companion animals: nosocomial infections as one reason for the rising prevalence of these potential zoonotic pathogens in clinical samples.. Int J Med Microbiol.

[5] Cuny C, Friedrich A, Kozytska S, Layer F, Nübel U (2010). Emergence of methicillin-resistant *Staphylococcus aureus* (MRSA) in different animal species.. Int J Med Microbiol.

[6] Mahoudeau I, Delabranche X, Prevost G, Monteil H, Piemont Y (1997). Frequency of isolation of Staphylococcus intermedius from humans.. J Clin Microbiol.

[7] Perreten V, Kadlec K, Schwarz S, Gronlund Andersson U, Finn M (2010). Clonal spread of methicillin-resistant *Staphylococcus pseudintermedius* in Europe and North America: an international multicentre study.. J Antimicrob Chemother.

[8] Ruscher C, Lübke-Becker A, Wleklinski CG, Soba A, Wieler LH (2009). Prevalence of Methicillin-resistant Staphylococcus pseudintermedius isolated from clinical samples of companion animals and equidaes.. Vet Microbiol.

[9] Cohn LA, Middleton JR (2010). A veterinary perspective on methicillin-resistant staphylococci.. J Vet Emerg Crit Care (San Antonio).

[10] Hanselman BA, Kruth SA, Rousseau J, Weese JS (2009). Coagulase positive staphylococcal colonization of humans and their household pets.. Can Vet J.

[11] Manian FA (2003). Asymptomatic nasal carriage of mupirocin-resistant, methicillin-resistant *Staphylococcus aureus* (MRSA) in a pet dog associated with MRSA infection in household contacts.. Clin Infect Dis.

[12] Nienhoff U, Kadlec K, Chaberny IF, Verspohl J, Gerlach GF (2009). Transmission of methicillin-resistant *Staphylococcus aureus* strains between humans and dogs: two case reports.. J Antimicrob Chemother.

[13] Vincze S, Paasch A, Walther B, Ruscher C, Lübke-Becker A (2010). Multidrug- and methicillin resistant *Staphylococcus pseudintermedius* as a cause of canine pyoderma: a case report.. Berl und Münch Tierärztl Wochenschr.

[14] Walther B, Wieler LH, Friedrich AW, Kohn B, Brunnberg L (2009). *Staphylococcus aureus* and MRSA colonization rates among personnel and dogs in a small animal hospital: Effect on nosocomial infections.. Berl Münch Tierarztl Wochenschr.

[15] Engemann JJ, Carmeli Y, Cosgrove SE, Fowler VG, Bronstein MZ (2003). Adverse clinical and economic outcomes attributable to methicillin resistance among patients with *Staphylococcus aureus* surgical site infection.. Clin Infect Dis.

[16] Ishihara K, Shimokubo N, Sakagami A, Ueno H, Muramatsu Y (2010). Occurrence and molecular characteristics of methicillin-resistant *Staphylococcus aureus* and methicillin-resistant *Staphylococcus pseudintermedius* in an academic veterinary hospital.. Appl Environ Microbiol.

[17] Weese JS, Rousseau J, Willey BM, Archambault M, McGeer A (2006). Methicillin-resistant *Staphylococcus aureus* in horses at a veterinary teaching hospital: frequency, characterization, and association with clinical disease.. J Vet Intern Med.

[18] Black CC, Solyman SM, Eberlein LC, Bemis DA, Woron AM (2009). Identification of a predominant multilocus sequence type, pulsed-field gel electrophoresis cluster, and novel staphylococcal chromosomal cassette in clinical isolates of mecA-containing, methicillin-resistant *Staphylococcus pseudintermedius*.. Vet Microbiol.

[19] Ruscher C, Lübke-Becker A, Semmler T, Wleklinski CG, Paasch A (2010). Widespread rapid emergence of a distinct methicillin- and multidrug-resistant *Staphylococcus pseudintermedius* (MRSP) genetic lineage in Europe.. Vet Microbiol.

[20] Chuang CY, Yang YL, Hsueh PR, Lee PI (2010). Catheter-related bacteremia caused by *Staphylococcus pseudintermedius* refractory to antibiotic-lock therapy in a hemophilic child with dog exposure.. J Clin Microbiol.

[21] Kempker R, Mangalat D, Kongphet-Tran T, Eaton M (2009). Beware of the pet dog: a case of *Staphylococcus intermedius* infection.. Am J Med Sci.

[22] Frank LA, Kania SA, Kirzeder EM, Eberlein LC, Bemis DA (2009). Risk of colonization or gene transfer to owners of dogs with meticillin-resistant *Staphylococcus pseudintermedius*.. Vet Dermatol.

[23] Loeffler A, Pfeiffer DU, Lloyd DH, Smith H, Soares-Magalhaes R (2010). Meticillin-resistant *Staphylococcus aureus* carriage in UK veterinary staff and owners of infected pets: new risk groups.. J Hosp Infect.

[24] Becker K, von Eiff C, Murray P, Baron E, Jorgensen J, Landry ML, Pfaller M (2011). Staphylococcus, Micrococcus and other catalase-positive cocci.. Manual of Clinical Microbiology, 10th ed.

[25] Merlino J, Watson J, Rose B, Beard-Pegler M, Gottlieb T (2002). Detection and expression of methicillin/oxacillin resistance in multidrug-resistant and non-multidrug-resistant *Staphylococcus aureus* in Central Sydney, Australia.. J Antimicrob Chemother.

[26] Bannoehr J, Franco A, Iurescia M, Battisti A, Fitzgerald JR (2009). Molecular diagnostic identification of *Staphylococcus pseudintermedius*.. J Clin Microbiol.

[27] Lina G, Piemont Y, Godail-Gamot F, Bes M, Peter MO (1999). Involvement of Panton-Valentine leukocidin-producing *Staphylococcus aureus* in primary skin infections and pneumonia.. Clin Infect Dis.

[28] Harmsen D, Claus H, Witte W, Rothganger J, Turnwald D (2003). Typing of methicillin-resistant *Staphylococcus aureus* in a university hospital setting by using novel software for *spa* repeat determination and database management.. J Clin Microbiol.

[29] Enright MC, Day NP, Davies CE, Peacock SJ, Spratt BG (2000). Multilocus sequence typing for characterization of methicillin-resistant and methicillin-susceptible clones of *Staphylococcus aureus*.. J Clin Microbiol.

[30] Bannoehr J, Ben Zakour NL, Waller AS, Guardabassi L, Thoday KL (2007). Population genetic structure of the *Staphylococcus intermedius* group: insights into *agr* diversification and the emergence of methicillin-resistant strains.. J Bacteriol.

[31] Futagawa-Saito K, Sugiyama T, Karube S, Sakurai N, Ba-Thein W (2004). Prevalence and characterization of leukotoxin-producing *Staphylococcus intermedius* in Isolates from dogs and pigeons.. J Clin Microbiol.

[32] Futagawa-Saito K, Makino S, Sunaga F, Kato Y, Sakurai-Komada N (2009). Identification of first exfoliative toxin in *Staphylococcus pseudintermedius*.. FEMS Microbiol Lett.

[33] Iyori K, Hisatsune J, Kawakami T, Shibata S, Murayama N (2010). Identification of a novel *Staphylococcus pseudintermedius* exfoliative toxin gene and its prevalence in isolates from canines with pyoderma and healthy dogs.. FEMS Microbiol Lett.

[34] van Duijkeren E, Kamphuis M, van der Mije IC, Laarhoven LM, Duim B (2011). Transmission of methicillin-resistant *Staphylococcus pseudintermedius* between infected dogs and cats and contact pets, humans and the environment in households and veterinary clinics.. Vet Microbiol.

[35] Wertheim HF, Melles DC, Vos MC, van Leeuwen W, van Belkum A (2005). The role of nasal carriage in *Staphylococcus aureus* infections.. Lancet Infect Dis.

[36] Lozano C, Gomez-Sanz E, Benito D, Aspiroz C, Zarazaga M (2011). *Staphylococcus aureus* nasal carriage, virulence traits, antibiotic resistance mechanisms, and genetic lineages in healthy humans in Spain, with detection of CC398 and CC97 strains.. Int J Med Microbiol.

[37] Rubin JE, Chirino-Trejo M (2010). Pharyngeal, rectal and nasal colonization of clinically healthy dogs with *Staphylococcus aureus*.. Vet Microbiol.

[38] Fazakerley J, Nuttall T, Sales D, Schmidt V, Carter SD (2009). Staphylococcal colonization of mucosal and lesional skin sites in atopic and healthy dogs.. Vet Dermatol.

[39] Aires de Sousa M, Crisostomo MI, Sanches IS, Wu JS, Fuzhong J (2003). Frequent recovery of a single clonal type of multidrug-resistant *Staphylococcus aureus* from patients in two hospitals in Taiwan and China.. J Clin Microbiol.

[40] Walther B, Monecke S, Ruscher C, Friedrich AW, Ehricht R (2009). Comparative molecular analysis substantiates a zoonotic potential of equine Methicillin- resistant *Staphylococcus aureus* (MRSA).. J Clin Microbiol.

[41] Paul NC, Moodley A, Ghibaudo G, Guardabassi L (2011). Carriage of Methicillin-Resistant S*taphylococcus pseudintermedius* in Small Animal Veterinarians: Indirect Evidence of Zoonotic Transmission.. Zoonoses and Public Health.

[42] Van Hoovels L, Vankeerberghen A, Boel A, Van Vaerenbergh K, De Beenhouwer H (2006). First case of *Staphylococcus pseudintermedius* infection in a human.. J Clin Microbiol.

[43] Soedarmanto I, Kanbar T, Ulbegi-Mohyla H, Hijazin M, Alber J (2011). Genetic relatedness of methicillin-resistant *Staphylococcus pseudintermedius* (MRSP) isolated from a dog and the dog owner.. Res Vet Sci.

